# A cross-sectional analysis about bacterial vaginosis, high-risk human papillomavirus infection, and cervical intraepithelial neoplasia in Chinese women

**DOI:** 10.1038/s41598-022-10532-1

**Published:** 2022-04-22

**Authors:** Xiaolin Xu, Yichan Zhang, Liqun Yu, Xingxian Shi, Min Min, Lijuan Xiong, Jia Pan, Peipei Liu, Guizhen Wu, Guolan Gao

**Affiliations:** 1 Savaid Medical School, University of Chinese Academy of Sciences, Beijing, China; 2 NHC Key Laboratory of Biosafety, Chinese Center for Disease Control and Prevention, National Institute for Viral Disease Control and Prevention, Beijing, China; 3 Department of Obstetrics and Gynecology, Aviation General Hospital, Beijing, China; 4 Department of Electrical and Computer Engineering, Johns Hopkins University, Baltimore, MD USA; 5 Department of Obstetrics and Gynecology, Peking University International Hospital, Beijing, China

**Keywords:** Microbiology, Microbial communities, Microbiome

## Abstract

Bacterial vaginosis (BV) is a genital infection that frequently presents in women infected with human papillomavirus (HPV), but the correlation between BV, HPV and cervical intraepithelial neoplasia (CIN) development is still elusive. We organized a cross-sectional analysis which enrolled 624 participants and obtained 423 samples of vaginal secretions from them, including 193 HPV-negative samples and 230 HR-HPV-positive samples. We used 16S rRNA sequencing to measure the vaginal microbiota diversity in women with different BV, HPV and CIN status, and then calculated risk factors for CIN by logistic regression. We found that the diversity of vaginal microbiota was significantly increased after BV, HPV and BV-infected CIN group. The Observed species and Chao1 index of H.C group showed little difference with normal group, while its Shannon index was considerable higher than normal group. *L. iners* enriched in HPV infection group compared with others significantly. BV (OR = 0.358; 95% CI = 0.195–0.656; *P* < .05) and HR-HPV infection (OR = 0.016; 95% CI = 0.004–0.072; *P* < .001) were risk factors for CIN. In conclusion, we consider BV as a risk factor for CIN. The enrichment of *L. iners* under HPV infection state may contribute to maintenance of vaginal dysbiosis, and BV infection could facilitate the disturb.

## Introduction

Cervical cancer affects millions of women worldwide, with an estimated incidence of 604,200 new cases and 310,000 deaths in 2020, with China having 109,700 (18.2%) new cases and 59,700 (17.6%) deaths^1^. The prevalence of cervical cancer in China remains higher than in the Western World due to lower vaccine coverage among young women and less awareness of regular cervical cancer screening in adult. Persistent human papillomavirus (HPV) infection causes cervical intraepithelial neoplasia (CIN) that may develop into cervical cancer if not detected in its early stages during screening or treated well once detected. According to a systematic review by Yin et al.^2^ the rate of high-risk HPV (HR-HPV) infection caused by types 16, 52, 58, 53, and 18 is up to 19.0% in mainland Chinese women. Infection with HR-HPV is an independent risk factor for CIN and cervical cancer. HPV infection is the most common sexually transmitted infection (STI) especially in women with multiple sex partners^3^. Other factors like smoking, contraception and antibiotic use can disrupt the clearance of HPV leading to a high viral load that can trigger cervical lesions formation^4^.

Bacterial vaginosis (BV) occurs in the reproductive age group with 15–50% of women presenting with vaginal discharge, peculiar smell, itching, or increased vaginal pH levels^3^. There is no standard definition of BV, but it is widely recognized that its development is a process characterized by the dysregulation of vaginal microbiota with increased levels of *Gardnerella vaginalis (G. vaginalis)*, *Atopobium*, *Prevotella*, *Sneathia*, *Peptostreptococcus*, *Megasphera*, BV-associated bacterium 1 (BVAB1) to BVAB3, and decreased levels of *Lactobacilli* spp.^5,6^. The classification of community state types (CSTs) summarizes five types of vaginal microbiota in healthy women based on different domination of bacterium. CST I, II, III, V are dominated by *Lactobacillus crispatus* (*L. crispatus*), *L. gasseri*, *L. iners,* and *L. jensenii,* respectively; whereas CST IV is defined as the depletion of *Lactobacilli* spp.^7–9^. Women with CST IV vaginal communities are more likely to experience BV, abortion, preterm labor, increased susceptibility to human immunodeficiency virus (HIV) and HPV infection, or delayed HPV infection clearance^5,10^. The percentage of women with CST IV cluster is significantly higher in Black and Hispanic women who also have a higher incidence of cervical cancer than that in Asian and White women^11^.

The association between BV and CIN was described 30 years ago, but the mechanism remains elusive. Some research revealed that BV is an independent risk factor for persistent HPV infection^12–15^. Greater microbiota species diversity is observed in women infected with HPV. The abundances of bacteria associated with BV like *Sneathia*, *Prevotella,* and *Megashaera* are significantly higher in women who are positive for HPV^6^. Previous research focused on clarifying the association between the cervical cytological lesion and BV due to inadequate technological development^16–18^. With the popularity of colposcopy, recent studies have relied mainly on biopsy under colposcopy, which is more accurate than a thin prep cytology test (TCT), to determine the stage of cervical lesions.

This study is focused on documenting the association of BV, CIN, and HR-HPV infection. We used 16S rRNA gene sequencing to display the vaginal microbiota in Chinese women and compared the composition of microbiota between women with different BV, HPV, or CIN statuses. We aimed to find a promising marker for vaginal dysbiosis.

## Results

### Population characteristics

Samples were collected from November 2020 to April 17, 2021, from a total of 624 enrolled participants. 201 participants were excluded because of vulvovaginal candidiasis (VVC), trichomonas vaginitis (TV), other STIs infection (complete exclusion criteria seen in “Methods” Section), or unqualified DNA concentration of secretions. A total of 423 samples were finally included in this experiment (Table 1). Detailed information was available in the Supplementary Table S1 online.

**Table 1:** Population characteristics by HPV status (total n = 423).

Variables	HPV- (n = 193), n (%)	HR-HPV + (n = 230), n (%)	${\chi }^{2}$ Value	*P* value *
Age (years)			13.033	0.001**
25–39	142 (73.58)	135 (58.70)		
40–54	32 (16.58)	72 (31.30)		
≥ 55	19 (9.84)	23 (10.00)		
Gestation (times)			1.551	0.671
0	35 (18.13)	33 (14.35)		
1	66 (34.20)	76 (33.04)		
2	67 (34.72)	90 (39.13)		
≥ 3	25 (12.95)	31 (13.48)		
Parturition (times)			0.235	0.889
0	39 (20.21)	46 (20.00)		
1	110 (56.99)	127 (55.22)		
≥ 2	44 (22.80)	57 (24.78)		
Number of sexual partners 15.715			0.000**	
1	190 (98.45)	204 (88.70)		
2	3 (1.55)	24 (10.43)		
≥ 3	0 (0.00)	2 (0.87)		
Contraception			15.249	0.002**
Condom	159 (82.38)	188 (81.74)		
IUD	29 (15.03)	18 (7.83)		
Hormonal contraceptive	1 (0.52)	1 (0.43)		
None	4 (2.07)	23 (10.00)		
Clinical status			72.22	0.000**
BV infection	54 (27.98)	106 (46.09)		
Multiple HPV infection	0 (0.00)	86 (37.39)		
CIN	0 (0.00)	94 (40.87)		

All variables were showed as frequencies and proportions. HPV−: HPV (human papillomavirus)-negative; HPV + : HR-HPV positive; IUD: intrauterine device; BV: bacterial vaginosis; CIN: cervical intraepithelial neoplasia.

* *P* values were calculated by Pearson Chi-squared ${( \chi }^{2})$ test. ** statistically significant, *P* < .05.

According to the recommendations of the American Cancer Society (ACS), women with a cervix should undergo routine cervical cancer screening at least every five years starting at age 25 years until 65 years. We selected women between the age of 25 and 65 years as our participants, and divided them into 3 groups (25–39, 40–54, ≥ 55 years). There was no significant correlation between HPV infection and times of gestation or parturition, but there were significant correlations between age, contraception, and number of sexual partners. Of the 423 samples obtained, 160 out of 423 (37.83%) of the women had BV infections. The HPV-negative group had significantly lower rates of BV infection than the HPV-positive group (*P* < 0.001). A total of 230 out of the 423 samples (54.37%) suffered HR-HPV and 86 out of the 230 samples (37.39%) had more than one type of HPV infection. Women with HR-HPV who progressed to CIN were 94 out of 230 (40.87%).

**Figure 1:**
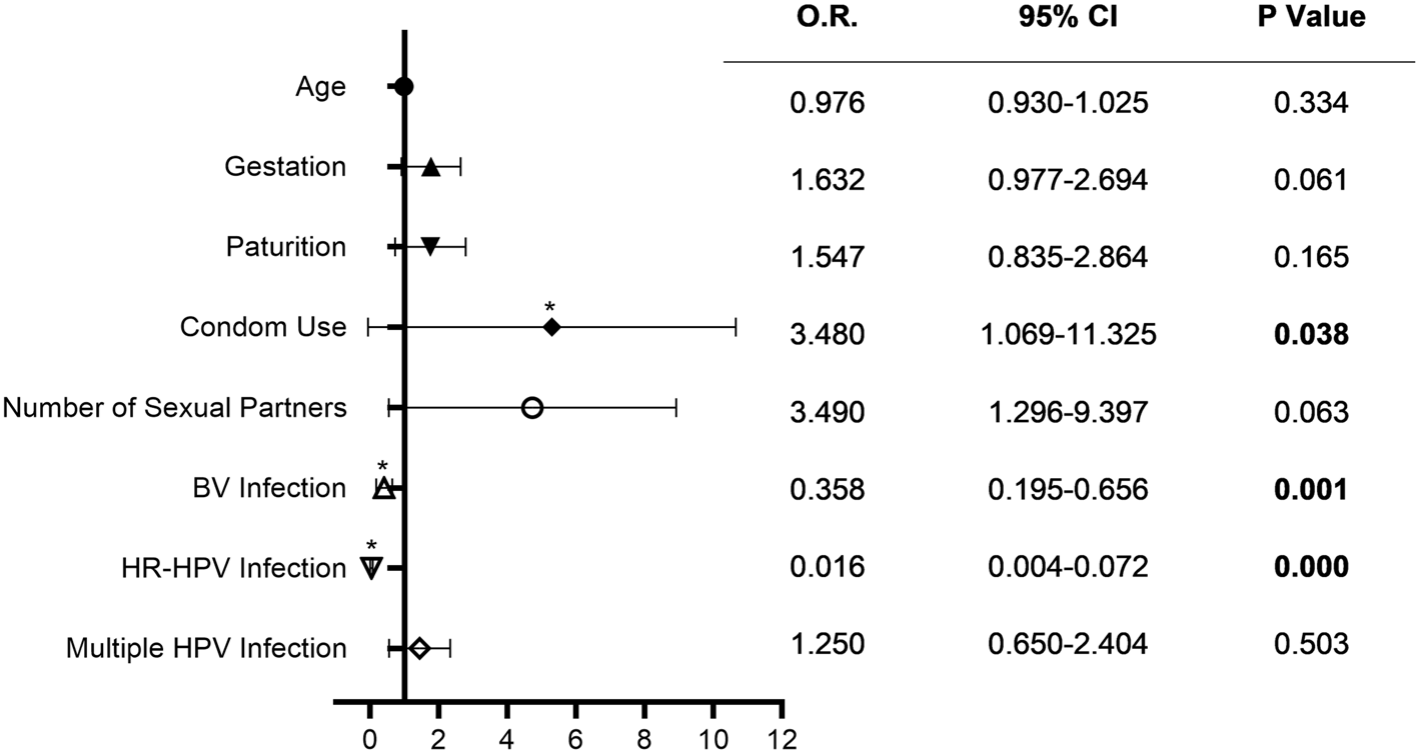
Odds ratio of possible risk factors for CIN Using Logistic regression. Bars represented 95% confidence intervals. *O.R.*: odds ratio, *CI*: confidence interval. * *P* < .05.

Next, we conducted logistic regression analysis on possible risk factors for CIN including age, times of gestation and parturition, methods of contraception, number of sexual partners, BV, HPV infection, and multitype HPV infection, which were collected in the questionnaire. As shown in Fig. 1, we found that condom use (OR = 3.480; 95% CI = 1.069–11.325; *P* < 0.05) was a protective factor for CIN, whereas BV (OR = 0.358; 95% CI = 0.195–0.656; *P* < 0.05) and HR-HPV infection (OR = 0.016; 95% CI = 0.004–0.072; *P* < 0.001) were risk factors for CIN.

### The difference in composition of vaginal microbiota in the HPV-negative and HPV-positive groups

To identify the difference in composition of vaginal microbiota between women with and without HPV infection, we sequenced the V3-V4 hypervariable region of the 16S rDNA gene utilizing 16S rRNA sequencing. We acquired 15,137 OTUs in our experiment, the sequence result was available in Supplementary Database 1 and the taxonomy profile was in Supplementary Table S2 online.

**Figure 2:**
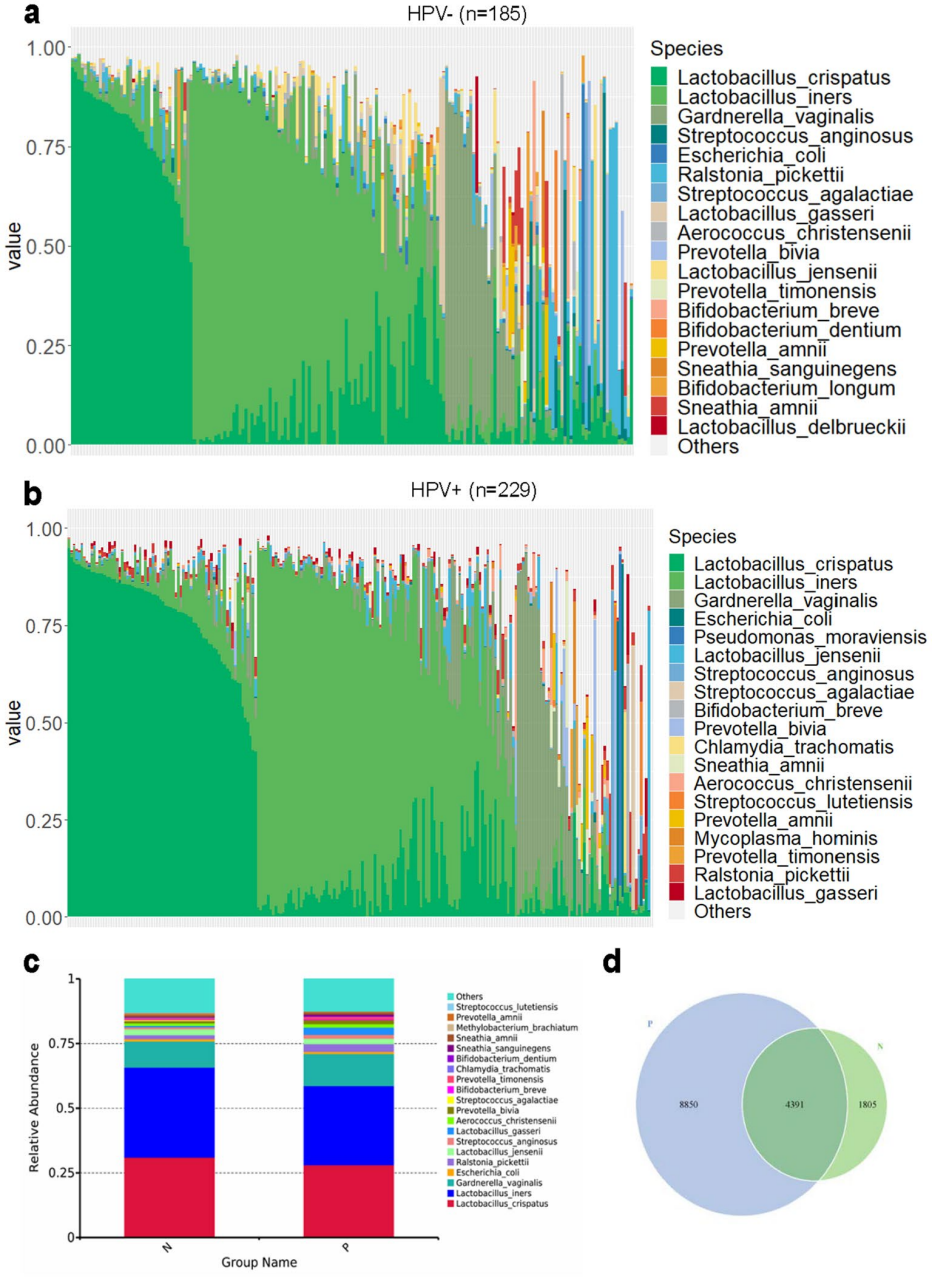
Vaginal microbiota composition in HPV-negative (HPV−) and HPV- positive (HPV +) group samples. (**a**). Relative abundance of the top 19 vaginal microbiota at the species level in HPV-negative group women. (**b**). Relative abundance of the top 19 vaginal microbiota at species level in HPV-positive group women. (**c**). Relative abundance bar chart for HPV-negative and HPV-positive groups at the species level. (**d**). Venn graph for HPV-negative and HPV-positive groups. Each circle represents a group, and the number of circles and overlapping parts represents the number of OTUs shared between the groups and the number of non-overlapping parts represents the number of unique OTUs of the group. P: HPV-positive; N: HPV-negative.

In the HPV-negative group, 8 out of 193 samples and 1 out of 230 samples in the HPV-positive group were excluded because of low DNA copy numbers. The relative abundance of the top 19 prevalent vaginal microbiota in HPV-negative and HPV-positive women samples with *Lactobacillus* spp. predominant in both groups are displayed in Fig. 2a, b. As shown in Fig. 2c, the abundance of *Lactobacillus* spp. in the HPV-negative group was much higher than that in the HPV-positive group. Meanwhile, microbiota composition in the HPV-positive group was more complicated. Bacteria such as *Gardnerella vaginalis*, *Ralstonia pickettii*, *Streptococcus anginosus*, *Prevotella bivia*, *Prevotella timonensis*, *Bifidobacterium dentium,* and *Sneathia sanguinegens* presented more frequently in the HPV-positive group. We analyzed the OTUs numbers in each group and displayed them in a Venn graph in Fig. 2d. The unique OTUs in the HPV-positive group were significantly higher than in the HPV-negative group. These data suggest that HPV infection could lead to an increase in vaginal microbiota complexity.

### The relative abundance of vaginal microbiota of different BV, HPV and CIN statuses

**Figure 3:**
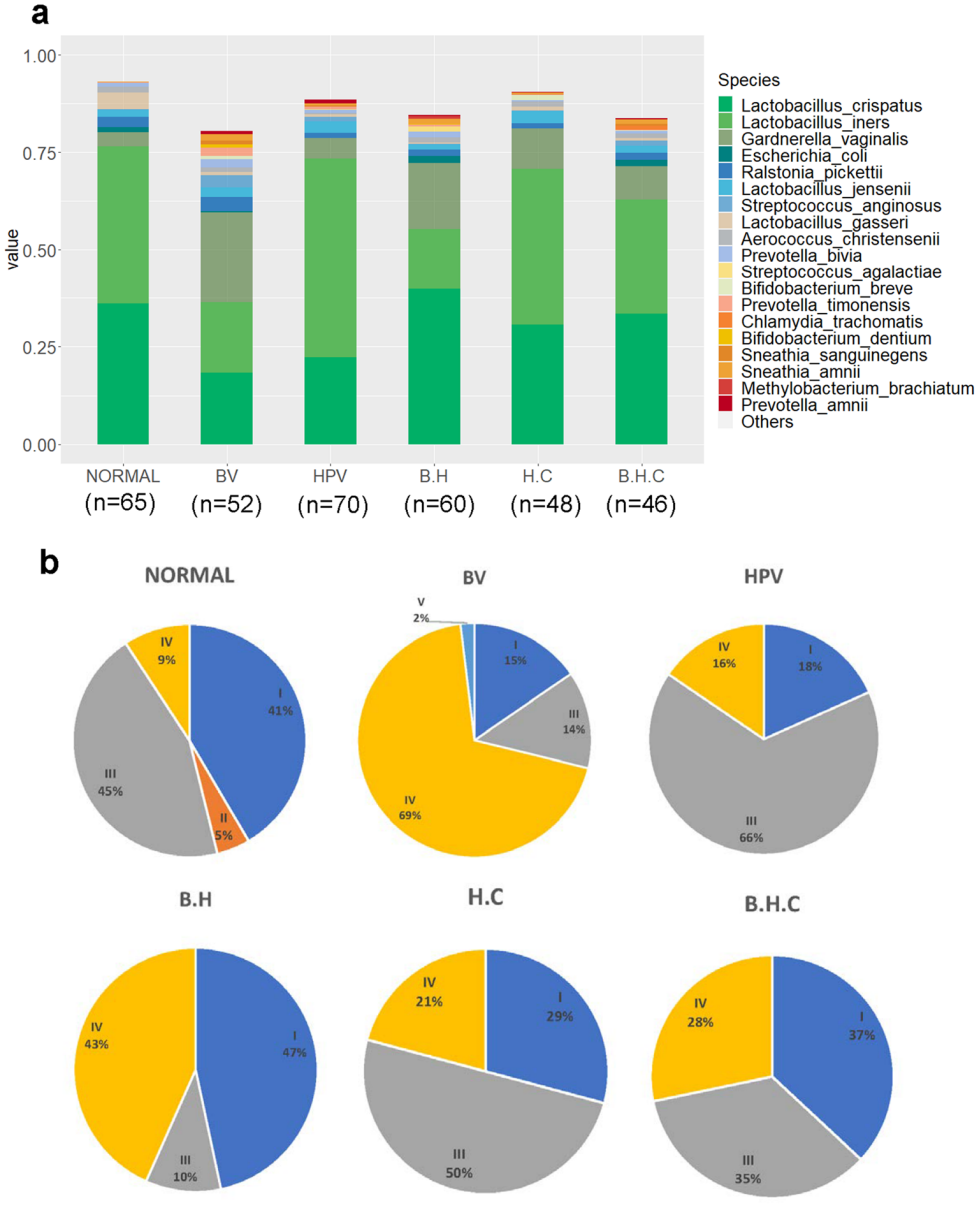
Relative abundance of vaginal microbiota of different BV, HPV, and CIN statuses. (**a**). Relative abundance of vaginal microbiota in 6 groups. (**b**). CST distribution of 6 groups. BV: BV infected; HPV: HPV infected without CIN; B.H: infected with BV and HPV; H.C: HPV infected with CIN; B.H.C: infected with BV, HPV, and CIN.

To further estimate the vaginal microbiota changes under different clinical condition, we divided all the samples into 6 groups according to the following BV, HPV or CIN statuses: Normal, BV (BV infection), HPV (HR-HPV infection), B.H (BV and HR-HPV co-infection), H.C (HR-HPV infection combined with CIN), and B.H.C (BV and HR-HPV co-infection combined with CIN). To better distinguish the differences between groups, we conducted random screening of the grouped data to balance the sample size of each group. A total of 342 samples were collected and the numbers of final samples of each group were 65, 52, 71, 60, 48, and 46, respectively (Supplementary Tables S3–S8 for detailed information). In Fig. 3a, we displayed the top 19 vaginal microbiota at the species level. *Lactobacillus* spp. had the highest percentage relative abundance of up to 82.6% in the normal group. Women with HPV infection only showed little change in the *Lactobacillus spp.* genera in their vaginal microbiota, but the percentage of *L.crispatus,* which presented most in a healthy vaginal environment was partly replaced by *L.iners*. *Lactobacillus* spp. lost its dominance (with less than 60% in composition) in the remaining three groups of women, who were all diagnosed with BV infection with a more complex bacterial composition.

We defined the community state types (CSTs) according to the dominant (> 60% of relative abundance in one sample) *Lactobacillus* species type. CST I, II, III, V were dominated by *L. crispatus*, *L. gasseri*, *L. iners,* and *L. jensenii,* respectively, whereas CST IV was defined as the depletion of *Lactobacillus* spp.^19^. In Fig. 3b, the CST II cluster was only shown in the normal group and CST V in the BV group. The transformation of CST I to CST III was most apparent in the HPV-positive group, which indicated that *L.iners* may be a key species during HPV infection. Women diagnosed with BV infection were more likely to be defined as being in the CST IV cluster. Interestingly, the percentages of CST IV cluster among BV and HPV co-infected women with CIN were significantly lower than those in the B.H group, and CST III cluster percentage was increased, which also indicates the importance of *L.iners.*

### Higher microbial diversity in women with BV and HPV co-infection

**Figure 4:**
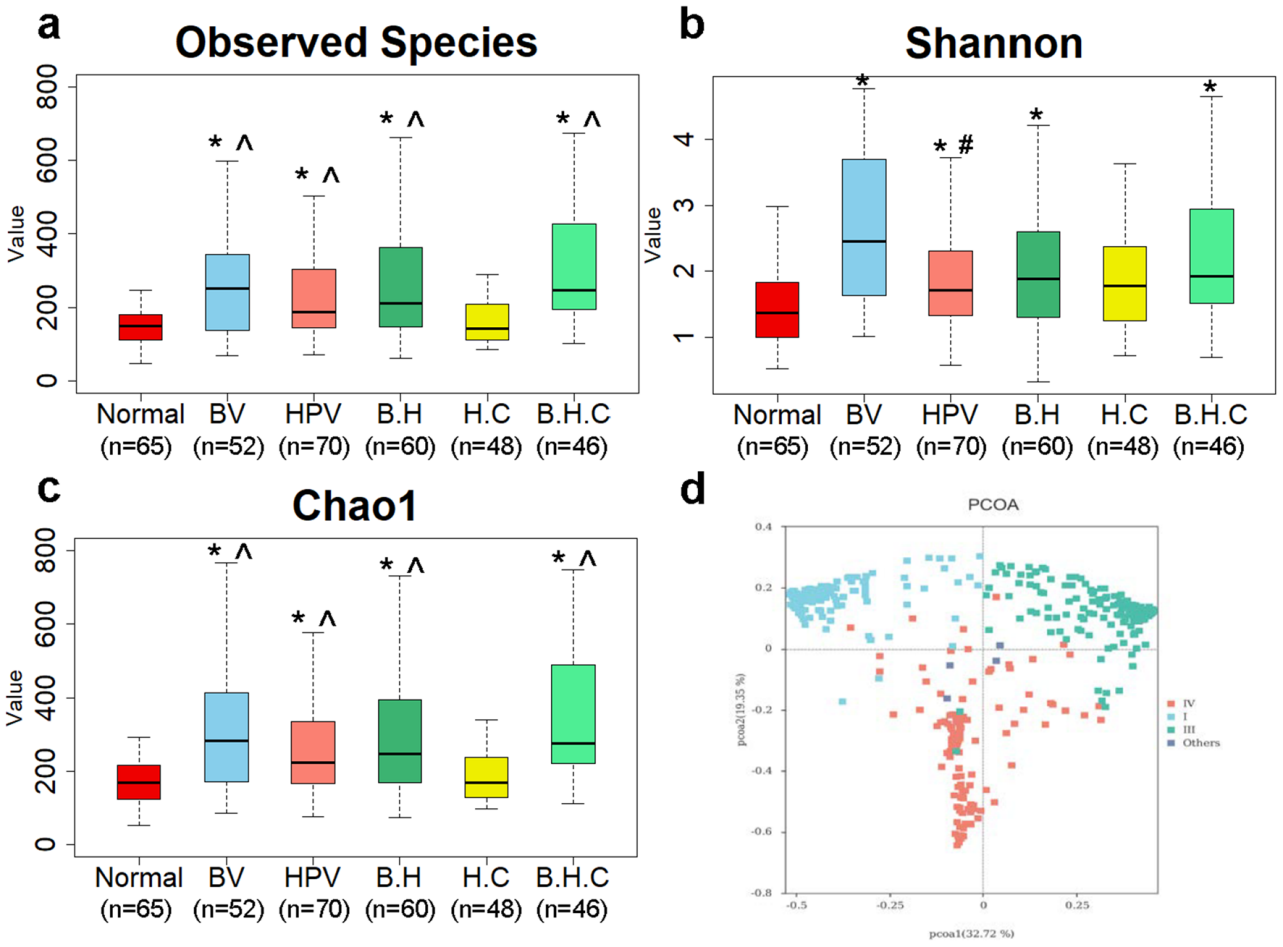
Microbial diversity in 6 groups. (**a**). Observed species indexes of samples in 6 groups. (**b**). Shannon index. (**c**). Chao1 index. (**d**) PCoA based on the Bray–Curtis dissimilarity in CST clusters. Each dot represented a sample of the corresponding group dyed with the same color. I, III, IV represented CST I, III, IV, and others represented samples in CST II and V clusters. BV: BV infected; HPV: HPV infected without CIN; B.H: infected with BV and HPV; H.C: HPV infected with CIN; B.H.C: infected with BV, HPV, and CIN. The *P* values in a-c were all calculated by the Wilcoxon rank-sum test, alpha value = 0.05. * Significantly different compared with the Normal group. *P* < .001. ^ Significantly different compared with the H.C group. *P* < .001. # Significantly different compared with the B.H.C group. *P* < .001.

The observed species count the number of visually observed species, Shannon’s diversity index and Chao1 index reflex the richness and evenness of species, respectively. These 3 indexes were used to measure the alpha diversity of microbiomes in 6 groups. High variability was observed among the samples; thus outliers were not shown in the boxplot (Fig. 4a–c). Three indexes of microbial diversity in the BV, HPV, B.H, and B.H.C groups were all significantly higher than those in the normal group, which indicated that either BV or HPV infection could disturb the balance of vaginal flora (*P* < 0.001). Besides, the observed species and the Chao1 index of the H.C group were lower than those of the BV, HPV B.H, and B.H.C groups (*P* < 0.001). The Shannon indexes for the HPV and H.C groups were significantly lower than that of the B.H.C group (*P* < 0.001). Interestingly, the Observed species and Chao1 index of H.C group showed little difference with normal group, while its Shannon index was higher than normal group. In other words, for women with CIN, the total amount of vaginal microbiome changed slightly, but the complexity substantial rising, and when co-infection occurred, the richness and evenness of bacteria increased significantly (*P* < 0.001).

The beta diversity was measured with the PCoA based on the Bray–Curtis dissimilarity. In Fig. 4d, all the samples were mainly separated into three groups related to CST clusters (*P* < 0.001). *P* values were calculated by the ANOSIM test. Biodiversity was not dependent on BV, HPV, or CIN status (Supplementary Fig. S1 online).

### Important phylotype in BV and HPV infection

**Figure 5:**
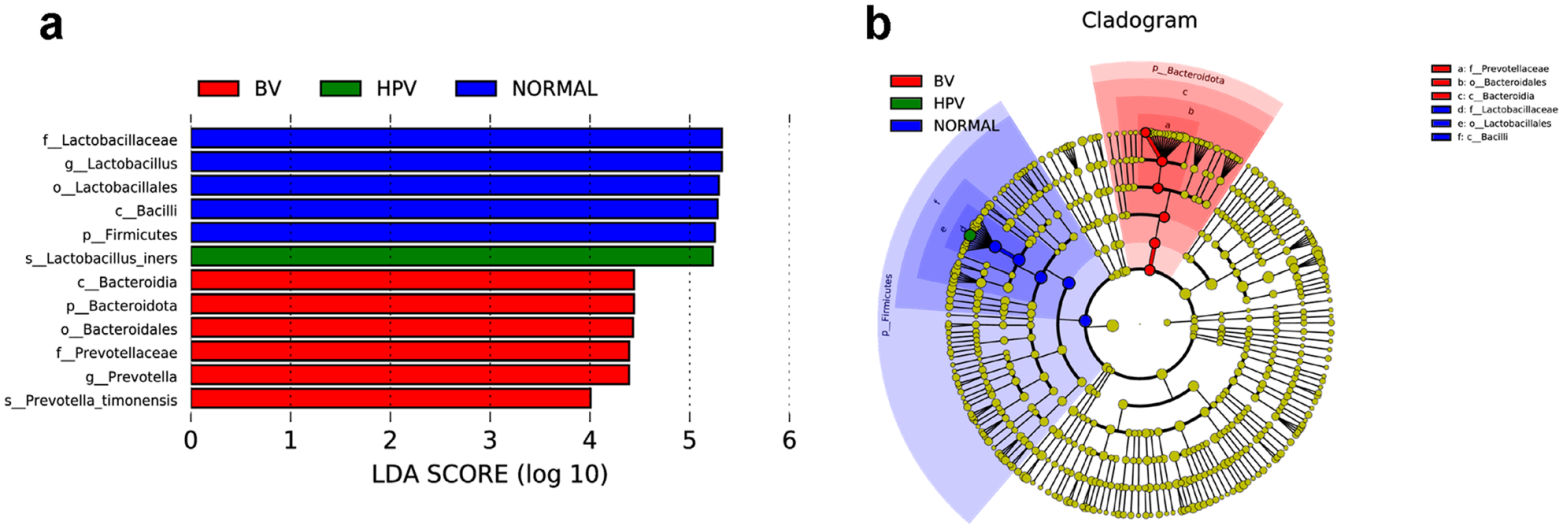
Metagenomic biomarker between 6 groups using LEfSe analysis. (**a**). Enrichment in bacterial taxa between 6 groups. The threshold for the LDA score was 4.0. Groups with no statistically significant species were not shown in the figure. (**b**). Cladogram for different bacteria between groups. Non-significant biomarkers are shown in yellow, and significantly different biomarkers are dyed the same color as the corresponding group. Alpha value for the Kruskal–Wallis rank sum test was 0.05.

To find out which bacteria are responsible for the community differences between 6 groups, we used LEfSe (Linear Discriminant Analysis Effect Size) to analyze the bacterial diversity and find biomarkers. It combines statistical difference analysis and the impact score of the species on the grouping results while emphasizing statistical significance and biological relevance. Figure 5a, b mainly showed the species with significant differences in linear discriminant analysis (LDA) score greater than 4.0, which is the biomarker with a statistical difference by Kruskal–Wallis rank sum test (Alpha value = 0.05). Like a previous study, we found a significant enrichment of *Lactobacillus* genera in the normal group compared with the group with BV or HPV-infected women^20^. *L.iners* presented most in HPV infected women and anaerobic bacteria *Prevotellaceae* family were enrichment when women infected with BV. No considerable biomarker for CIN was found.

### Gene functional pathways of bacterial taxa associated with BV and HPV infection

**Figure 6:**
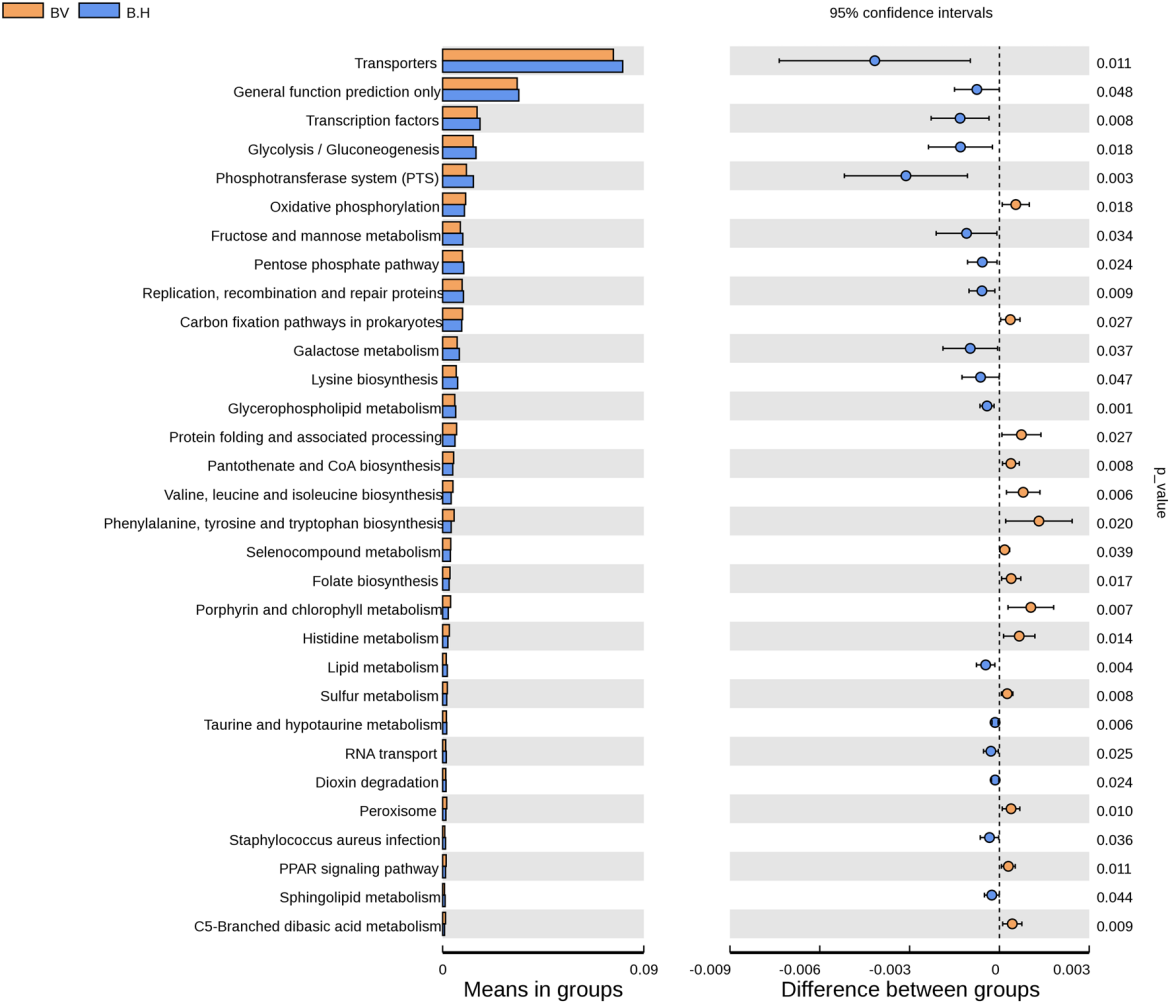
Predicted bacterial function pathways differences between BV and BV + HPV co-infected women. PICRUSt algorithm was used to predict bacterial gene functions from the KEGG annotated databases^22–24^. The bar chart on the left represents the percentage of the abundance of a certain metabolic pathway from all metabolic pathways in the two groups of samples. Corrected *P* values are on the right side. T-test (two-tailed) was used to calculate the *P-*value. *P* value less than 0.05 was considered statistically significant.

To better understand the bacterial function on HPV infection among BV infected women, we also explored the microbiota function using PICRUSt (Phylogenetic Investigation of Communities by Reconstruction of Unobserved States)^21^. Gene we detected were matched with the KEGG (Kyoto Encyclopedia of Genes and Genomes) database, predicted raw data was available in Supplementary Database 2. The KEGG pathway we tested was presented in Supplementary Fig. S2^22–24^. 31 kinds of pathways changed in B.H compared with BV (*P* < 0.05). Pathways related to transporters, transcriptional factors, phosphotransferase system (PTS), carbohydrate metabolism (glycolysis/gluconeogenesis, galactose metabolism, fructose and mannose metabolism), pentose phosphate pathway, replication, recombination and repair proteins, glycerophospholipid metabolism, lysine biosynthesis, lipid metabolism, , taurine and hypotaurine metabolism, RNA transport, dioxin degradation, Staphylococcus aureus infection and sphingolipid metabolism were enriched in the BV and HPV co-infected women, whereas oxidative phosphorylation, carbon fixation pathways in prokaryotes, protein folding and associated processing, pantothenate and CoA biosynthesis, protein biosynthesis (valine, leucine, isoleucine, phenylalanine, tyrosine and tryptophan), selenocompound metabolism, folate biosynthesis, porphyrin and chlorophyll metabolism, histidine metabolism, sulfur metabolism, peroxisome, PPAR signaling pathway and C5-Branched dibasic acid metabolism were depleted after HPV infection (Fig. 6).

## Discussion

Studies on the association of vaginal microbiota, HPV infection, and CIN have been a hotspot since gene sequencing technology was developed. Vaginal microbiota changes such as depletion of *Lactobacillus* spp. and increased microbiota diversity are a well-accepted finding in women infected with HPV^25^. We found that the vaginal microbiota in women with BV or HPV infection was more complex and had a reduction of *Lactobacillus* spp. (Fig. 2). This is in line with the current research trends in China and abroad^5,7,9,10^. Condom use (OR = 3.480; 95% CI = 1.069–11.325; *P* < 0.05) was a protective factor, whereas BV (OR = 0.358; 95% CI = 0.195–0.656; *P* < 0.05) and HR-HPV infection (OR = 0.016; 95% CI = 0.004–0.072; *P* < 0.001) were risk factors for CIN in our survey (Fig. 1) and other research studies too^4,26–28^. The prevalence of smoking and hormonal contraceptive use in Chinese women is lower than in western countries, so we could not make any correlation between them and CIN in our cohort.

This study was a cross-sectional analysis based on a large sample size of Chinese women focused on the association of vaginal microbiota disturbance and HPV infection. A surprising finding in our survey was the CST cluster distribution. CST II and V rarely appeared in our cohort especially after HPV infection regardless of BV condition (Fig. 3b), which was not consistent with foreign studies done in White, Hispanic, and Black women^11,29,30^. This may be because of the ethnic and geographic diversity^31^. The distribution of CST IV cluster in women with CIN was slightly lower than expected probably due to women with severe BV or VVC in whom a colposcopy was not done to prevent the spread of infection^32,33^. Drugs used to treat the vaginitis may have inhibited the colonization of many bacteria, thus the relative abundance of BV associated bacteria and microbiota diversity in the H.C or B.H.C group (Figs. 3a and 4) was slightly lower than in the BV group. The actual relative abundance and diversity could be higher than the available data, but we still could conclude that BV-free women (H.C group) have significantly lower vaginal microbiota diversity than the B.H.C group (Fig. 4b). Cervical lesion may cause a considerable change in the complexity of microbiota (Fig. 4b), and inhibit the growth of bacteria taxa (Fig. 4c), so that the Shannon and Chao1 index in H.C group is opposite.

Additionally, women infected with HPV have a higher proportion of *L.iners* than healthy women, which led us to focus on the characteristics of this special *Lactobacillus* spp. Similar to the previous studies, we found that *L. iners* was not only present in the vaginal ecosystem of healthy women, but also of those in a transitional stage or BV state^19,34,35^. Comparative analysis LEfSe between 6 groups also presented the significant enrichment of *L.iners* in HPV group (Fig. 5). One of the possible reasons for the considerable increasing of *L.iners* in HPV group and decreasing in H.C and B.H.C group (Fig. 3a) is that the different morphology and Gram-staining properties of *L.iners* with other *Lactobacillus* spp. shown by genome analyses^36–39^. Non-H_2_O_2_ or lactic acid producing *Lactobacillus* species *L.iners* could inhibit other *Lactobacillus* species growth, facilitate the multiply of anaerobic bacteria and maintain vaginal dysbiosis^35^. Furthermore, in 2011, Macklaim et al.^40^ used whole-genome sequencing to define the smallest species of *L. iners*, *L. iners* AB-1, which is present in both healthy women and those treated with antimicrobials. One of the reasons for its persistence in the vaginal epithelia despite the development of BV and treatment with antibiotics is the presence of fibronectin (Fn)-binding adhesins^41^. The percentage of samples dominated with *L. iners* was higher in the B.H.C group than in the B.H group (Fig. 3b), which makes us question whether *L. iners* AB-1 was present in our sample. Further research is necessary to detect *L. iners* AB-1 in abnormal vaginal microbiota flora.

Our study was a cross-sectional analysis conducted in one hospital, which may have limited the observation of the dynamic changes of vaginal microbiota after HPV infection and CIN progression. Plenty of factors could have an impact on the long-lasting process from HPV infection to CIN. Without longitudinal observation, it is hard to conclude the causal relationship between vaginal microbiota and CIN progression. In addition, we chose two specialists to diagnose BV to minimize the misdiagnosis rate, but it is hard to avoid missing the diagnosis of asymptomatic or molecular BV and reporting bias. We only performed 16S rRNA gene sequencing in our study, which failed to distinguish *L. iners* AB-1 from *L. iners* in samples. Therefore, further study is needed to better understand vaginal microbiota changes in HPV persistence and CIN progression.

The strengths of this study include the large sample size, which was enough to organize the samples into 6 groups. We also did functional prediction of bacteria, which provides a reference for further mechanism research. We found that BV and HPV co-infected women did not have a higher diversity of vaginal microbiota unless CIN occurred. For future studies, short-, medium- and long-term follow-ups are needed to observe dynamic changes of the vaginal microbiota and the disease process in women with BV or HPV infection. Further investigation is also needed to understand the characteristics of *L. iners* in both healthy and unhealthy states.

In conclusion, BV was considered as a risk factor for CIN. Cervical lesion may cause a considerable change in the complexity of microbiota, and inhibit the growth of bacteria taxa. BV and HPV infection could trigger an increase in vaginal microbiota diversity, especially BV, which could decrease *Lactobacillus* spp. domination. HPV infection could significantly increase the abundance of *L. iners*, which may contribute to maintain vaginal dysbiosis, and BV infection could facilitate the disturb.

## Methods

### Participant enrollment

This study was approved by the Ethics Committee of the Aviation General Hospital, Beijing, China (Ethical approval No. 2021-KY-01-02). Written informed consent was obtained from all participants and all methods were performed in accordance with the guidelines and regulations. Women attending the Aviation General hospital, located in Beijing, China were recruited in our study. The close-ended questionnaire was drafted by two of the authors and conducted face-to-face. Prior to actual distribution, a pilot was conducted with 20 participants, and the results of pilot questionnaire were not included in subsequent analysis. The inclusion criteria included: (a.) age range from 25 to 65 years; (b.) untreated BV; (c.) voluntary signing of informed consent form and questionnaire; (d.) had sexual experience. The exclusion criteria included: (a.) current pregnancy; (b.) undergoing menstrual period; (c.) vaccinated against HPV; (d.) sexual activity, vaginal irrigation or drug application performed 48 h before sampling; (e.) diagnosed with vulvovaginal candidiasis (VVC), trichomonas vaginitis, or other STIs like *Neisseria gonorrhoeae*, *Chlamydia trachomatis*, human immunodeficiency virus (HIV), hepatitis B virus (HBV) or *Treponema pallidum*; (f.) accompanied by hypertension, diabetes, immune system disease or other diseases; and (g.) antibiotics use in the past 30 days.

### Sample collection

Participants were put in the lithotomy position on the gynecological examination bed after signing the informed consent form and finishing the questionnaire. A well-trained clinical doctor swabbed the vagina using two disposable sterile swabs to collect cervical and vaginal secretions that were then stored in 2 ml saline and 2 ml phosphate buffer saline (PBS, HyClone, USA) tubes, respectively. Samples were kept on ice and transferred to the laboratory for subsequent testing.

### BV, HPV, and CIN diagnosis

An experienced doctor made a diagnosis of BV if a patient met three out of the following four Amsel’s criteria: (a.) increased, thin, and homogeneous vaginal discharge; (b.) vaginal pH greater than 4.5; (c.) presence of clue cells; and (d.) amine odor when potassium hydroxide (KOH) was added to the vaginal secretions. The Hybrid Capture 2 assay (HC2) was used to detect seventeen HR-HPV types (16, 18, 31, 33, 35, 39, 45, 51, 52, 53, 56, 58, 59, 66, 68, 73, 82) and six low-risk types (6, 11, 42, 43, 44, 81). Women who were positive for HR-HPV also accepted TCT to define the cytological lesion. If the results were atypical squamous cells of undetermined significance (ASC-US) or more severe lesions such as low-grade squamous intraepithelial lesions (LSIL) or high-grade squamous intraepithelial lesions (HSIL), they were recommended for colposcopy to define the grade of CIN.

### DNA extraction

FastDNA Spin Kit (MP Biomedicals, USA) was used for DNA isolation from vaginal secretions. We added 200 μl of the samples suspended in saline and 1 ml cell lysis solution (CLS-TC) to Lysing Matrix A (FastPrep) and then followed the protocol. DNA concentration and purity was monitored on 1% agarose gel. According to the concentration, DNA was diluted to 1 ug/ μl using sterile water.

### 16S rRNA gene sequencing

V3-V4 hypervariable fragments of the 16S rRNA gene were amplified using primers 338F (338F: 5’- ACTCCTACGGGAGGCAGCA -3’) and 806R (806R: 5’- GGACTACHVGGGTWTCTAAT -3’) by PCR with the barcode. Mixture PCR products was purified with Qiagen Gel Extraction Kit (Qiagen, Germany). TruSeq DNA PCR-Free sample preparation kits (Illumina, USA) were used for library construction. The library quality was assessed on the Qubit@2.0 Fluorometer (Thermo Scientific) and Agilent Bioanalyzer 2100 system. At last, the library was sequenced on an Illumina NovaSeq platform (Illumina, CA, USA) and 250 bp paired-end reads were generated.

### Sequence analysis

Paired-end reads was assigned to samples based on their unique barcode and truncated by cutting off the barcode and primer sequence. Paired-end reads were merged using FLASH (V1.2.7, http://ccb.jhu.edu/sofeware/FLASH)^42^. Quality filtering on the raw tags were performed under specific filtering conditions to obtain the high-quality clean tag according to the QIIME (V1.9.1, http://qiime.org/scripts/split libraries fastq.html) quality controlled process^43,44^. The tags were compared with the reference database (Silva database, https://www.arb-silva.de/) using UCHIME algorithm (UCHIME Algorithm, http://www.drive5.com/usearch/manual/uchime_algo.html) to detect chimera sequences, and then the chimera sequences were removed^45,46^. Then the Effective Tags finally obtained. Sequences analyses were performed by Uparse software (Uparse V7.0.1001, http://drive5.com/uparse/)^47^. Sequences with ≥ 97% similarity were assigned to the same OTUs. For each representative sequence, the Silva Database (http://www.arb-silva.de/) was used based on Mothur algorithm to annotate taxonomic information^48^. To study phylogenetic relationship of different OTUs and the difference of the dominant species in different samples, multiple sequence alignment were conducted using the MUSCLE software (Version 3.8.31, http://www.drive5.com/muscle/)^49^.

Alpha diversity indices Observed-species, Chao1 and Shannon were calculated with QIIME (V1.7.0) and displayed with R software (V3.6.3). Beta diversity was calculated by QIIME software (V1.9.1). Principal Coordinate Analysis (PCoA) analysis was displayed by WGCNA package, stat packages and ggplot2 package in R software. Linear Discriminant Analysis Effect Size (LEfSe) analysis and Phylogenetic Investigation of Communities by Reconstruction of Unobserved States (PICRUSt) were performed using the Novomagic, a free online platform for data analysis (https://magic.novogene.com), and the images were drawn by R^22–24,50^.

### Statistical analysis

All statistical analyses were calculated using SPSS version 23 (IBM, New York, NY) and R software (V3.6.3). The categorical variables collected in the questionnaire were displayed by frequencies and proportions. Statistical analyses were performed with Pearson’s Chi-squared ${( \chi }^{2})$ test. Logistic regression was used to calculate the value of odds ratio (OR) and assess the risk factors of CIN. *P* value less than 0.05 was considered statistically significant. The continuous variables in Observed species, Shannon and Chao1 index were not normally distributed. Thus, Wilcoxon rank-sum test was used to analyze the significant difference between groups (Alpha value = 0.05). *P* values of PCoA based on the Bray–Curtis dissimilarity were calculated by the ANOSIM test. LEfSe analysis was used to discover the metagenomic biomarker. Kruskal–Wallis rank-sum test and pairwise Wilcoxon test was calculate by LEfSe software (version 1.0). Alpha value less than 0.05 was considered statistically significant^50^. In PICRUSt part, two-tailed T-test was used to calculate the *P*-value. *P* value less than 0.05 was considered statistically significant.

## Supplementary Information

Supplementary Information 1.

Supplementary Information 2.

Supplementary Information 3.

Supplementary Information 4.

## Data Availability

All data generated or analyzed in this study are included in this published article and the Supplementary Information.
