# Supplementary material for: A cross-sectional analysis about bacterial vaginosis, high-risk human papillomavirus infection, and cervical intraepithelial neoplasia in Chinese women
Source: Sci Rep. 2022 Apr 22;12:6609. doi: 10.1038/s41598-022-10532-1 (PMC9033840; doi:10.1038/s41598-022-10532-1)
Supplement: Supplementary file 3 — Supplementary Information 3. [file 41598_2022_10532_MOESM3_ESM.pdf]

A Cross-sectional Analysis about Bacterial Vaginosis, High-risk Human Papillomavirus Infection, and Cervical Intraepithelial Neoplasia in Chinese Women

Xiaolin Xu[a,b,1], Yichan Zhang[a,b,1], Liqun Yu[c], Xingxian Shi[c], Min Min[c], Lijuan Xiong[c], Jia Pan[d], Peipei Liu[b,*], Guizhen Wu[b,*], Guolan Gao[a,e,*]

a Savaid Medical School, University of Chinese Academy of Sciences, Beijing, China

b NHC Key Laboratory of Biosafety, National Institute for Viral Disease Control and Prevention, Chinese Center for Disease Control and Prevention, Beijing, China

c Department of Obstetrics and Gynecology, Aviation General Hospital, Beijing, China

d Department of Electrical and Computer Engineering, Johns Hopkins University, Baltimore, MD, US

e Department of Obstetrics and Gynecology, Peking University International Hospital, Beijing, China

[*] Corresponding author: P.L. (email: liupp@ivdc.chinacdc.cn), G.W. (email: wugz@ivdc.chinacdc.cn) or G.G. (email: gaoguolan@ucas.ac.cn)

[1]These authors contributed equally to this work.

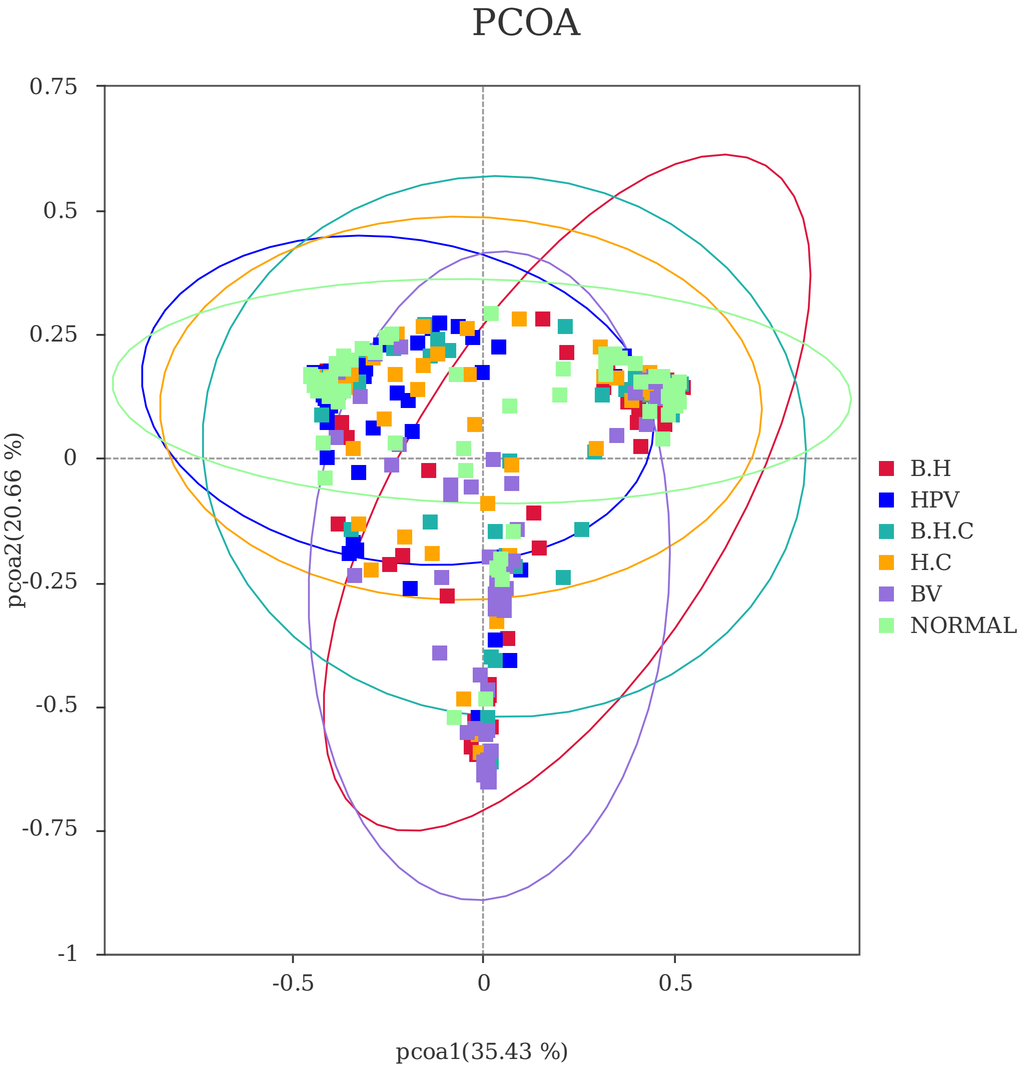

Supplementary Figure S1. PCoA based on the Bray-Curtis dissimilarity under different clinical status. Each dot represented a sample of the corresponding group dyed with the same color. The circles represented the overall trend line for each group. None of the samples in 6 groups showed separation tendency. BV: BV infected; HPV: HPV infected without CIN; B.H: infected with BV and HPV; H.C: HPV infected with CIN; B.H.C: infected with BV, HPV, and CIN.

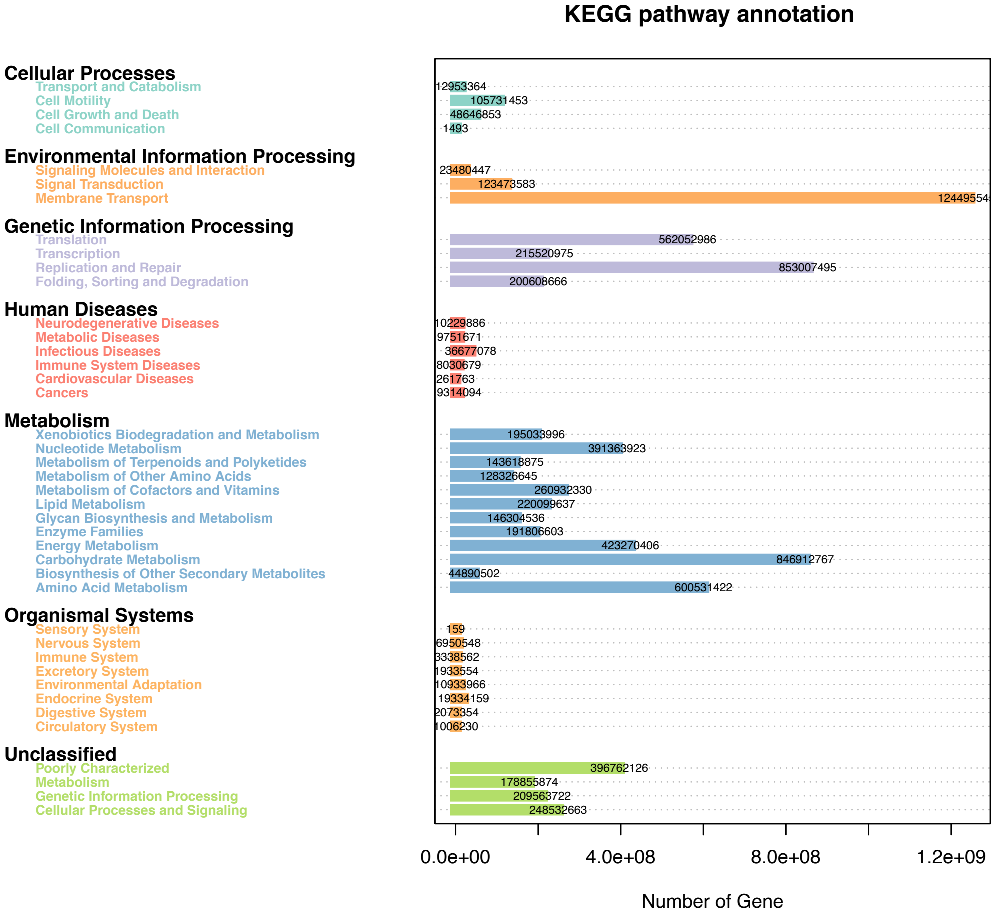

Supplementary Figure S2. Bacterial metagenomes functional prediction matched with KEGG database. Gene functional pathway of bacterial taxa were predicted using PICRUSt. Left side of the picture showed the KEGG pathway at level 1 and level 2, and the right side was number of gene contained in our sample.
